# The histone deacetylase inhibitor PCI-24781 as a putative radiosensitizer in pediatric glioblastoma cell lines

**DOI:** 10.1186/s12935-016-0306-5

**Published:** 2016-04-18

**Authors:** Pamela Viani de Andrade, Augusto Faria Andrade, Rosane Gomes de Paula Queiroz, Carlos Alberto Scrideli, Luiz Gonzaga Tone, Elvis Terci Valera

**Affiliations:** Department of Pediatrics, Ribeirão Preto Medical School, Hospital das Clínicas da Faculdade de Medicina de Ribeirão Preto-USP, University of São Paulo, 7º andar. Av. Bandeirantes, 3900, Bairro Monte Alegre, Ribeirão Preto, SP CEP 14048-900 Brazil; Department of Genetics, Ribeirão Preto Medical School, University of São Paulo, Av. Bandeirantes, 3900, Bairro Monte Alegre, Ribeirão Preto, SP CEP 14048-900 Brazil

**Keywords:** Glioblastoma, Radiation, Epigenetics, HDAC inhibitors, Double-strand DNA breaks

## Abstract

**Background:**

Glioblastoma (GBM) is considered to be one of the most aggressive tumors of the central nervous system (CNS). Even with the use of modern treatment protocols, the prognosis remains reserved, with children with GBM having a mean survival of 12–15 months.

**Methods:**

In the present study we investigated the potential radiosensitizing effect of PCI-24781, a potent pan-histone deacetylase inhibitor (HDACi), on the SF188 and KNS42 cell lines of pediatric GBM. Cell proliferation rates, clonogenicity and apoptosis were compared in the presence and absence of treatment with PCI-24781. We also compared the clonogenicity rates of the irradiated SF188 and KNS42 cell lines with or without previous treatment with PCI-24781 at the doses of 0.25–16 μM. In addition, we investigated the effects of PCI-24781 on the expression of some of the main proteins responsible for the repair of double-strand DNA breaks caused by irradiation.

**Results:**

The inhibitor blocked cell proliferation, induced death by apoptosis and reduced the colony forming capacity of the cell lines, both of them showing a significant decrease of colony formation at all irradiation doses. The expression of the Rad51 protein, important for the homologous recombination (HR) repair pathway, and of the DNA-PKcs, Ku70 and Ku86 proteins, important for the non-homologous end joining (NHEJ) repair pathway, was more reduced when the irradiated cell line was previously treated with PCI-24781 than when it was treated exclusively with radiotherapy.

**Conclusions:**

These findings demonstrate that HDACi PCI-24781 has a radiosensitizing profile that compromises the repair of double-strand DNA breaks in cells of pediatric GBM treated with radiotherapy.

## Background

Glioblastoma (GBM) is considered to be the most aggressive and most frequent central nervous system (CNS) tumor among adults, representing about 65 % of cases [1] in this population. In children, this is a relatively rare, although also very aggressive tumor, representing approximately 3 % of all childhood CNS neoplasms. Due to this low incidence, studies about pediatric GBM are less frequent than studies on adults [2].

The treatment strategy for GBM has not changed substantially over the last few years and essentially consists of surgical resection, radiotherapy and chemotherapy. Chemotherapy is usually employed to inhibit the replication of tumor clones or to provoke DNA damage in order to induce cell apoptosis [3, 4]. This multimodal therapy has been used as a standard treatment protocol for most patients, although survival continues to be low [5]. Five-year survival is reached by only 10 % of pediatric patients with GBM [6].

Temozolomide is the chemotherapeutic agent most frequently used for the treatment of adults and children with GBM but has been found to be poorly effective for the treatment of pediatric patients [7–9]. In addition to chemotherapy, radiotherapy is used for treatment after surgical resection in children older than 3 years. In children younger than 3 years, adjuvant chemotherapy is frequently used in an attempt to postpone or even to avoid radiotherapy [10]. However, both chemotherapy and radiotherapy are potentially toxic and contribute to significant morbidity and mortality [11].

Radiotherapy induces various types of DNA damage, among them double-strand breaks (DSB). DSB are defined as two simultaneous breaks in the opposite strands of the DNA helix. Although the cells are able to adapt to low levels of irreparable DNA damage, only one DSB is already potentially cytotoxic and can induce apoptosis in certain cell types [12–14]. The response of normal cells to DSBs includes the detection and repair of these radioinduced injuries by proteins sensing DNA damage and by DNA repair proteins, respectively. DSB repair in DNA occurs through two important repair pathways. One is homologous recombination (HR), which is the more precise mechanism of DSB repair and whose absence can lead to extensive genomic rearrangements and consequently to genomic instability. In HR, a homologous DNA sequence in a sister chromatid is used as a model for the repair of the broken strand [15]. The other is repair by nonhomologous end joining (NHEJ), which is a less precise form of DSB repair since the two endings of the broken DNA molecule are processed in order to form compatible endings that will be directly bound without being based on an undamaged DNA molecule. This mechanism is subject to a higher occurrence of errors since it does not require sequence homology for repair to occur, with the possible occurrence of small deletions or insertions [16].

New treatment strategies have been recently explored for gliomas, such as drug and therapy combinations directed at the molecular characteristics and genomic profile of the patients [17]. These studies include drugs that control epigenetic changes, which are of considerable interest as targets for cancer treatment because of their vital function in the cell processes that lead to oncogenesis [18]. The main epigenetic mechanisms explored in brain tumors are DNA methylation, the action of small noncoded RNAs and the modifications of histone proteins which determine the structure and activity of different chromatin regions and which are involved in cell memory and identity [19–22].

Among the various types of histone modifications, acetylation is the one that has been best characterized and that plays an important role in the modulation of the expression of genes that act on cell cycle control and contribute to the development and progression of neoplasia [20, 23–26]. Histone acetylation is performed by enzymes called histone acetyltransferases (HATs) which add acetyl radicals to the lysine residues of histone proteins, resulting in chromatin decompaction and transcriptional activity. On the other hand, histone deacetylases (HDACs) act by removing acetyl radicals and by recruiting corepressor complexes, resulting in chromatin compaction and gene silencing [23, 27, 28].

The objective of the present study was to assess the therapeutic potential of the HDAC inhibitor PCI-24781 against the pediatric GBM cell lines SF188 and KNS42, by analyzing the rates of cell proliferation, the clonogenic capacity and the apoptosis rates. We also investigated the effects of PCI-24781 as a possible radiosensitizing agent on both GBM lines by analyzing the clonogenic capacity and potential of the drug in changing the expression of proteins involved in the repair of double-strand DNA breaks caused by irradiation, i.e., Rad51, the heterodimer Ku70/Ku86 and DNA-PKcs.

## Methods

### Cell lines and culture conditions

The pediatric GBM lines SF188 (kindly provided by Dr. Nada Jabado and Dr. Damien Faury—McGill University—Canada) and KNS42 (obtained from the Japanese Collection of Research Bioresources Cell Bank through the Cell Bank of Rio de Janeiro) were used in the present study. The SF188 cell line was cultured in 75 cm^2^ bottles using HAM F10 culture medium supplemented with 60 mg/L penicillin, 100 mg/mL streptomycin and 10 % (v/v) fetal calf serum (FCS; Gibco BRL, Life Technologies, Carlsbad, CA, USA), pH 7.2–7.4, in a moist atmosphere containing 5 % CO_2_ at 37 °C. The KNS42 cell line was cultured in MEM medium supplemented with 60 mg/L penicillin, 100 mg/mL streptomycin and 5 % (v/v) FCS (Gibco BRL, Life Technologies), pH 7.2–7.4, in a moist atmosphere containing 5 % CO_2_ at 37 °C. The SF188 line originated from an 8-year-old male patient, has a *TP53* mutation in codon 266 [GGA(Gly)/GAA(Glu)], and is not tumorigenic. The KNS42 line originated from a 16-year-old male patient with primary GBM located in the right frontoparietal lobe and has a *TP53* mutation in codon 342[GGA(Arg)/TGA(Stop)] [29].

### Histone deacetylase inhibitor and treatments

According to the literature and to pilot experiments performed, a stock solution of the histone deacetylase inhibitor PCI-24781 (Selleckchem, Houston, TX, USA) was prepared at a final concentration of 50 mM in dimethylsulfoxide (DMSO; Mallinckrodt Chemical Works, St Louis, MO, USA) and stored in aliquots at −80 °C. Working solutions of 10 mM were prepared also in DMSO and stored in aliquots at −80 °C.

The drug was added to the culture medium and homogenized before being added to the cell culture. The DMSO concentration was 0.1 %. All controls were normalized by adding the same amount of DMSO as used for the treated cells.

### Cell proliferation assay

Cell proliferation was determined by the assay using the Resazurin Cell Viability kit according to manufacturer instructions. A total number of 2 × 10^3^ cells were seeded in 96-well plates and kept under culture conditions for 24 h. Next, the cells were treated with HDACi at the concentrations of 0.5, 1, 2, 4, 8 and 16 µM and incubated for 24, 48, 72 and 96 h. A resazurin solution was added to the plate (10 % of the initial volume in the well) at each treatment interval. The plates were incubated for 4 h under standard culture conditions. The non-fluorescent blue reagent is reduced to highly fluorescent resorufin by the dehydrogenase enzymes present in metabolically active cells with an absorbance peak at 570 nm. Absorbance readings were taken with the iMax Microplate Reader (Bio-Rad, Hercules, CA, USA), with the value detected being proportional to the quantity of cells in proliferation. These data were used to obtain the IC50 and IC30 values, which are defined as the concentrations necessary for a 50 and 30 % reduction of proliferation, respectively, using the Calcusyn software (Biosoft, Ferguson, MO, USA). Three independent experiments were performed in triplicate.

### Apoptosis assay

The assay for the detection of cell death was carried out by labeling apoptotic cells with annexin V fluorescein isothiocyanate (BD Biosciences Pharmigen, San Jose, CA, USA) and necrotic cells with propidium iodide (PI). Annexin V is a molecule with high affinity for phosphatidyl serine, to which it binds specifically. Phosphatidyl serine is a phospholipid present on the inner surface of the cell membrane which is externalized during the process of apoptosis and acts as a signal for the cells to be removed. Labeling with PI indicates that cells have lost their membrane integrity.

After a 48 h treatment at concentrations of 2, 4, 8 and 16 µM of the PCI-24781 inhibitor, the cells were trypsinized, centrifuged at 1000 r.p.m. for 5 min, washed with ice-cold 1 X PBS and resuspended in 300 μL 1 X annexin V binding buffer (BD Biosciences Pharmingen, San Jose, CA, USA). The cells were then labeled with 5 μL annexin V and 50 μL of a 50 μM PI solution, and analyzed with a BD FACSCalibur™ flow cytometer (BD Biosciences Pharmigen) for a total of 10,000 events per treatment. The values represent the mean and standard deviation of three independent experiments performed in triplicate.

### Clonogenic assay

The effect of the PCI-24781 inhibitor on clonogenic capacity was assessed according to the protocol of Franken et al. [30]. Cell suspensions of the SF188 and KNS42 lines were seeded at a density of 300 cells/well on six-well plates. After 24 h of incubation, the cells were treated with PCI-24781 concentrations of 0.25, 0.5, 1 and 2 µM and incubated in an oven at 37 °C for 48 h. Next, the culture medium was removed, the cells were washed with 1 X PBS, and drug-free medium was added in order to permit colony growth for approximately 7–10 days at 37 °C. After this period, the culture medium was removed, the cells were washed with PBS, fixed in absolute methanol and stained with 1 % Giemsa. Colonies of at least 50 cells were counted with a magnifying glass.

IC30 values, defined as the concentration necessary for a 30 % reduction of cell proliferation calculated with the Calcusyn software (Biosoft, Ferguson, MO, USA), were used for the assay combining the inhibitor with radiotherapy. The cells were then seeded as described earlier and treated with PCI-24781 for 48 h. Next, the cells were washed with 1X PBS and drug-free medium was added before irradiation with an RS-2000 X-Ray Irradiator Biological System (Rad Source Technologies, Inc., Suwanee, USA). The irradiation rate was 1115 Gy/min and the doses were 0, 0.5, 1, 2 and 4 Gy. The irradiated cells were then incubated at 37 °C for 7–10 days, fixed in absolute methanol, stained with Giemsa, and counted. The values represent the mean and standard deviation of three independent experiments carried out in triplicate.

### Protein expression—western blotting

#### Protein extraction

Total protein extraction was performed using RIPA^®^ lysis buffer (Sigma Aldrich Co., Saint Louis, MO, USA) together with protease and phosphatase inhibitors according to manufacturer instructions. Protein concentration was then determined by the method of Bradford, 1976 [31] using bovine serum albumin (BSA, 0.1 mg/mL) as standard. Absorbance readings at 595 nm wavelength were obtained with an iMax Microplate Reader spectrophotometer (Bio-Rad Laboratories Inc., CA, USA).

#### Western blotting

The methodology proposed by Sambrook et al. 1989 [32] was modified as described below. Equal concentrations (60 μg) of total proteins were submitted to 10 % polyacrylamide SDS gel electrophoresis using the Mini Protean II Dual Slab Cell system (Bio-Rad, USA). After transfer to a nitrocellulose membrane, the immunodetection process was started by blocking the membranes in a 5 % non-fat milk solution in 0.1 % TBST for one h. Next, the membranes were incubated overnight with the specific primary antibodies for each protein and then with anti-GAPDH and anti-β-actin (Santa Cruz Biotechnology, USA) for 1 h. The membranes were then washed with TBST (five washes of 5 min each), incubated with the appropriate horseradish peroxidase-conjugated secondary antibody and submitted to an additional wash cycle. The reaction was developed using the chemiluminescent substrate ECLTM (Amersham GE Healthcare, Buckinghamshire, UK) and visualized with the ChemiDOC XRS instrument (Bio-Rad, USA).

### Statistical analysis

All assays were carried out in triplicate and in three independent experiments. The mean and standard deviation of the experiments was considered for analysis by one-way ANOVA and two-way ANOVA followed by the nonparametric Bonferroni test.

The effect of the HDACi PCI-2478 on the combination and the effect of the irradiation dose on percent colony numbers was tested by logistic regression.

All analyses were performed with the aid of the SPSS 20.0 software (SPSS, Chicago, IL, USA), with the level of significance set at p < 0.05.

Percent cell proliferation, clonogenic survival, radiosensitization and apoptosis are presented graphically in the form of histograms using the GraphPad Prism software, version 5.0. Protein quantitation was performed using the Image J software, version1.49t (Research Services Branch, National Institute of Mental Health, Bethesda, MD, USA).

## Results

### Effects of the PCI-24781 inhibitor on cell proliferation

**Fig. 1:**
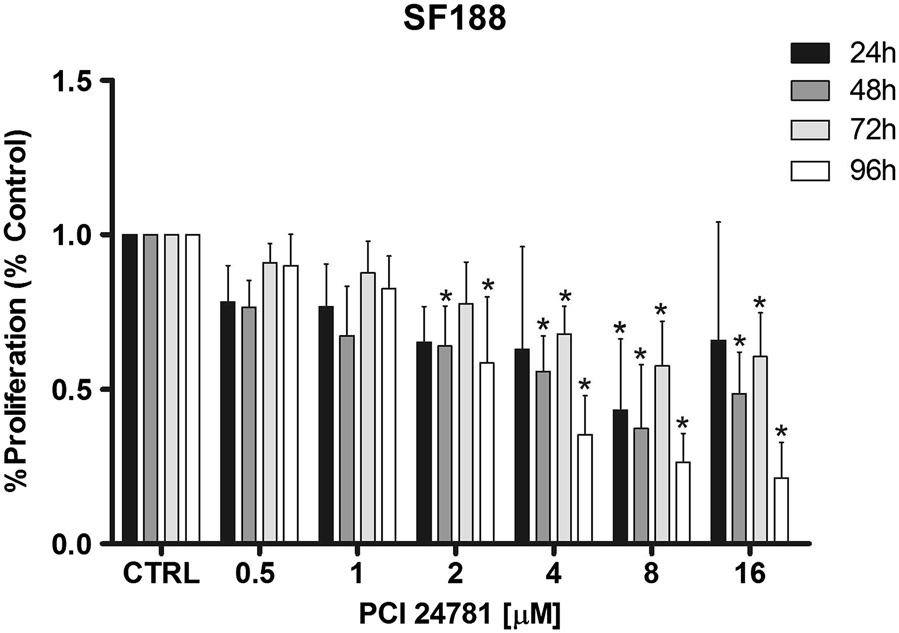
Analysis of cell proliferation of the SF188 line after treatment with PCI-24781. *p < 0.05 for treated cells compared to control

**Fig. 2:**
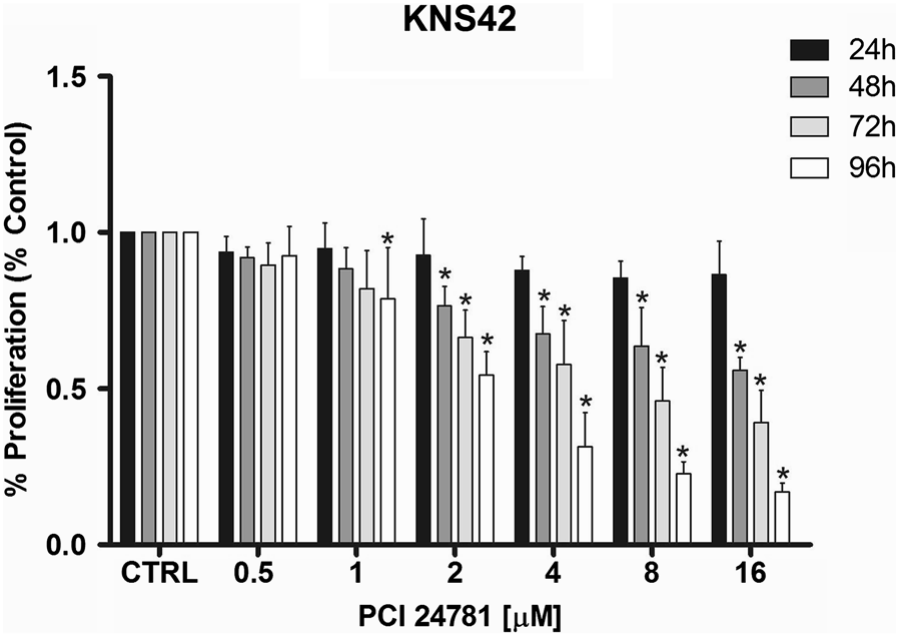
Analysis of cell proliferation of the KNS42 line after treatment with PCI-24781. *p < 0.05 for treated cells compared to control

We first investigated the effect of PCI-24781 on cell proliferation at doses of 0.5, 1, 2, 4, 8 and 16 µM for periods of 24, 48, 72 and 96 h on the cell lines SF188 and KNS42. A significant growth inhibition (p < 0.05) was detected compared to control at 48 and 96 h of treatment starting with the 2 µM dose. For the 72 h time point, this inhibition occurred starting with the 4 µM dose. After 24 h of exposure, the only dose that significantly reduced proliferation compared to control was 8 µM (Fig. 1). For the KNS42 line, a significant growth inhibition (p < 0.05) compared to control was observed at the doses of 2, 4, 8 and 16 µM at the 48 and 72 h time points and at the 96 h time point starting with the 1 µM dose, There was no significant inhibition at the 24 h time point (Fig. 2).

### Effects of the PCI-24781 inhibitor on the induction of apoptosis

**Fig. 3:**
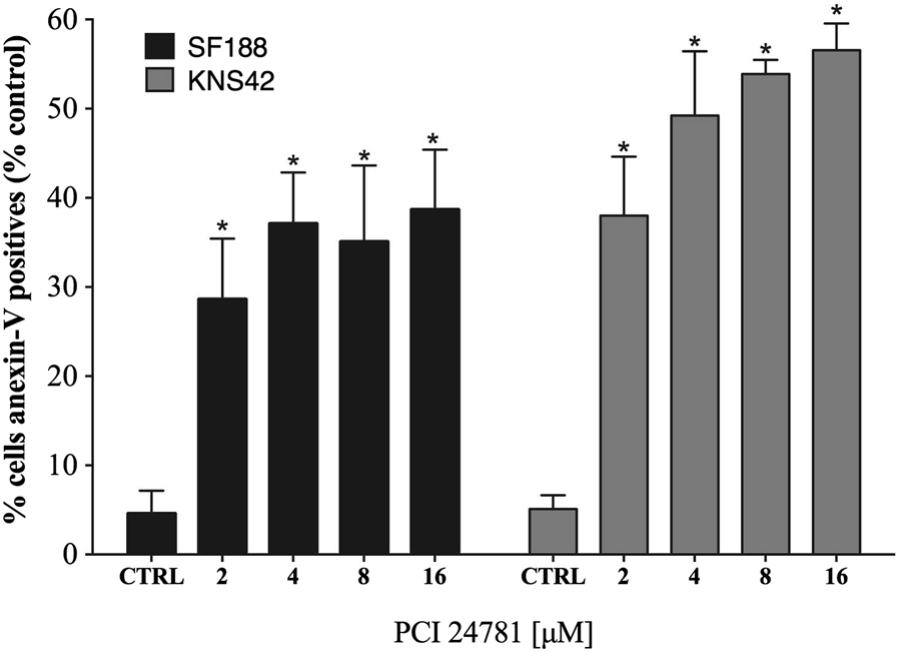
Percentage of annexin V-positive cells after treatment with PCI-24781 in the SF188 and KNS42 lines. *p < 0.05 for treated cells compared to control

We next investigated whether the reduction of cell proliferation observed was due to cell death by apoptosis. In order to determine if PCI-24781 induced apoptosis in cell lines of pediatric GBM we determined the percentage of apoptotic cells after labeling with annexin-V and propidium iodide (PI). Apoptosis was induced at 48 h at the doses of 2, 4, 8 and 16 µM in the pediatric GBM lines SF188 and KNS42. All doses tested showed a significant effect of increased apoptosis compared to control (p < 0.05). Starting at the 2 µM dose, the effect on the cell death rate of both lines was already quite significant compared to control, demonstrating that only a small dose of the PCI-24781 inhibitor was able to induce apoptosis. The cell death rate by apoptosis was almost 40 % for the SF188 line and 55 % for the KNS42 line at the maximum dose tested (Fig. 3).

### Effects of the PCI-24781 inhibitor on clonogenic capacity

**Fig. 4:**
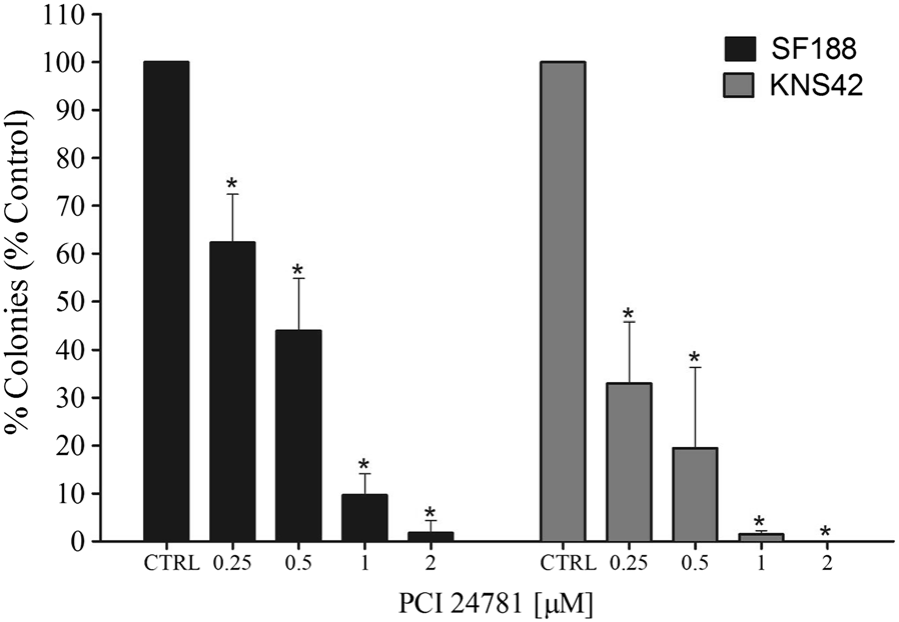
Percentage of colonies after treatment with PCI-24781 in the SF188 and KNS42 lines. *p < 0.001 for treated cells compared to control

In order to determine whether the reduction of proliferation affected cell growth in the long term and to test the capacity of tumor renewal, the test of clonogenic capacity was performed at the 48 h time point at the doses of 0.25, 0.5, 1 and 2 µM on the pediatric GBM lines SF188 and KNS42. The analysis revealed that all doses induced a significant fall in colony formation compared to control (p < 0.001). The SF188 line exhibited a reduction of colony formation of almost 99 % and the KNS42 line was unable to form colonies at the maximum dose used (Fig. 4).

### Radiosensitizing effect of the PCI-24781 inhibitor on clonogenic capacity

**Fig. 5:**
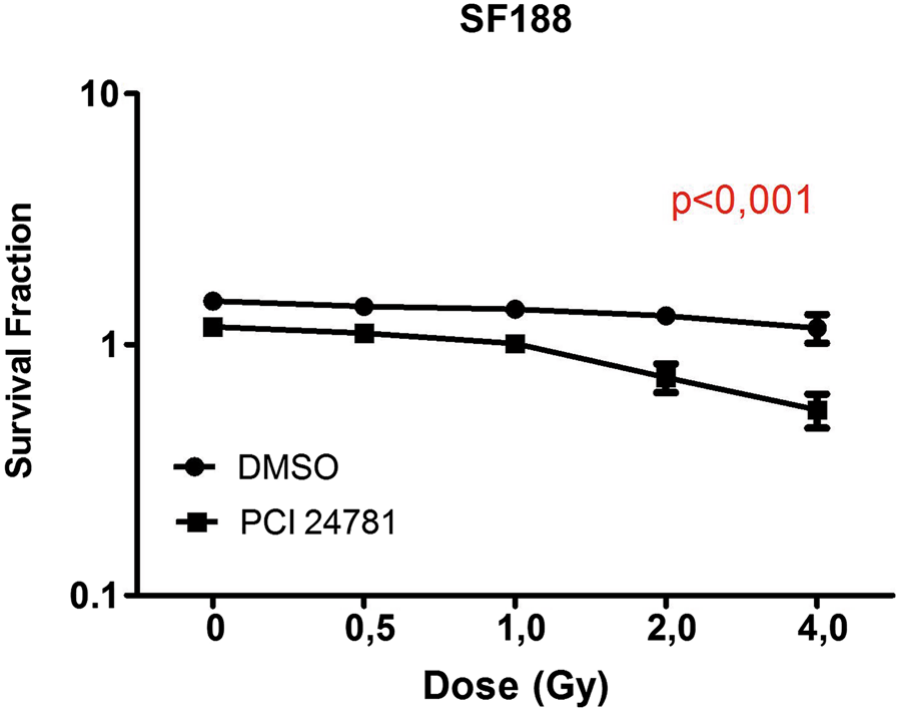
PCI-24781 radiosensitizes the SF188 line. *p < 0.001. Data are reported as the mean ± standard deviation of three independent experiments. *p* values obtained by linear regression are shown for all cell lines (DMSO versus PCI-24781)

**Fig. 6:**
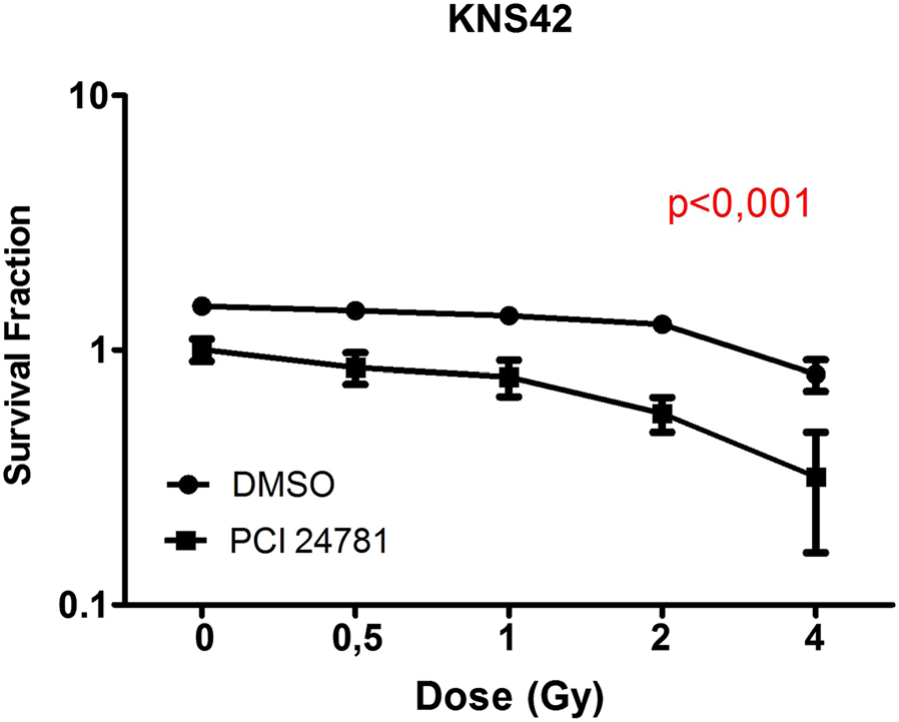
PCI-24781 radiosensitizes the KNS42 line. *p < 0.001. Data are reported as the mean ± standard deviation of three independent experiments. *p* values obtained by linear regression are shown for all cell lines (DMSO versus PCI-24781)

In order to determine the radiosensitizing effect of PCI-24781, the SF188 and KNS42 lines were treated with IC30 of the inhibitor, which was 0.25 µM for SF188 and 0.15 µM for KNS42, in combination with radiation at the doses of 0.5, 1, 2 and 4 Gy. It was observed that treatment with the inhibitor radiosensitized both cell lines, with a greater effect on the KNS42 line. The SF188 line showed a practically absent reduction of colony formation in the treatment with irradiation alone and a 45 % reduction with the combined treatment with PCI-24781 and the highest irradiation dose (Fig. 5). The KNS42 line showed a 20 % reduction of colony formation with irradiation alone and a 70 % reduction with the combined treatment with PCI-24781 and the highest irradiation dose (Fig. 6). Thus, treatment with PCI-24781 was able to sensitize strongly both lines to radiation.

### Effect of the PCI-24781 inhibitor on the expression of proteins responsible for the repair of double-strand DNA breaks

Western-Blot was used to determine the ability of the PCI-24781 agent to change the expression of some proteins present in the major pathways responsible for the repair of the DSB induced by irradiation: the RAD51 protein, important for the HR pathway, the Ku70/Ku86 heterodimer and the DNA-PKcs protein, important for NHEJ. For the analysis, we used the IC30 value (0.25 µM) of PCI-24782 for the SF188 line for 48 h and irradiation doses of 4 and 6 Gy. The proteins were collected after 4 h of exposure to irradiation.

**Fig. 7:**
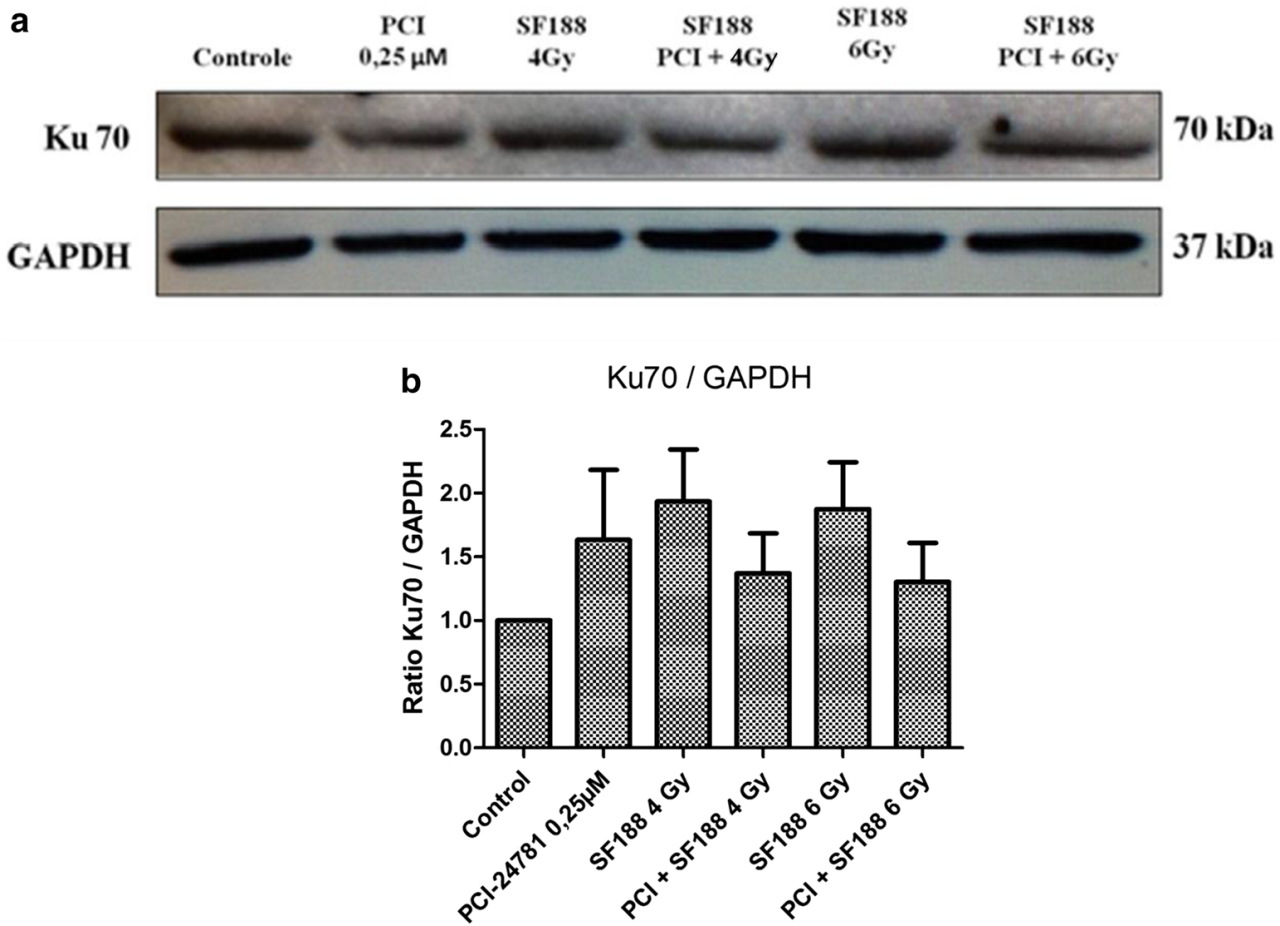
PCI-24781 alters the expression of the Ku70 protein. **a** PCI-24781 reduces the expression of the Ku70 protein, important for the repair of double-strand breaks caused by irradiation through the nonhomologous end joining (NHEJ) pathway. **b** Ratio between the Ku70 protein and endogenous GAPDH

**Fig. 8:**
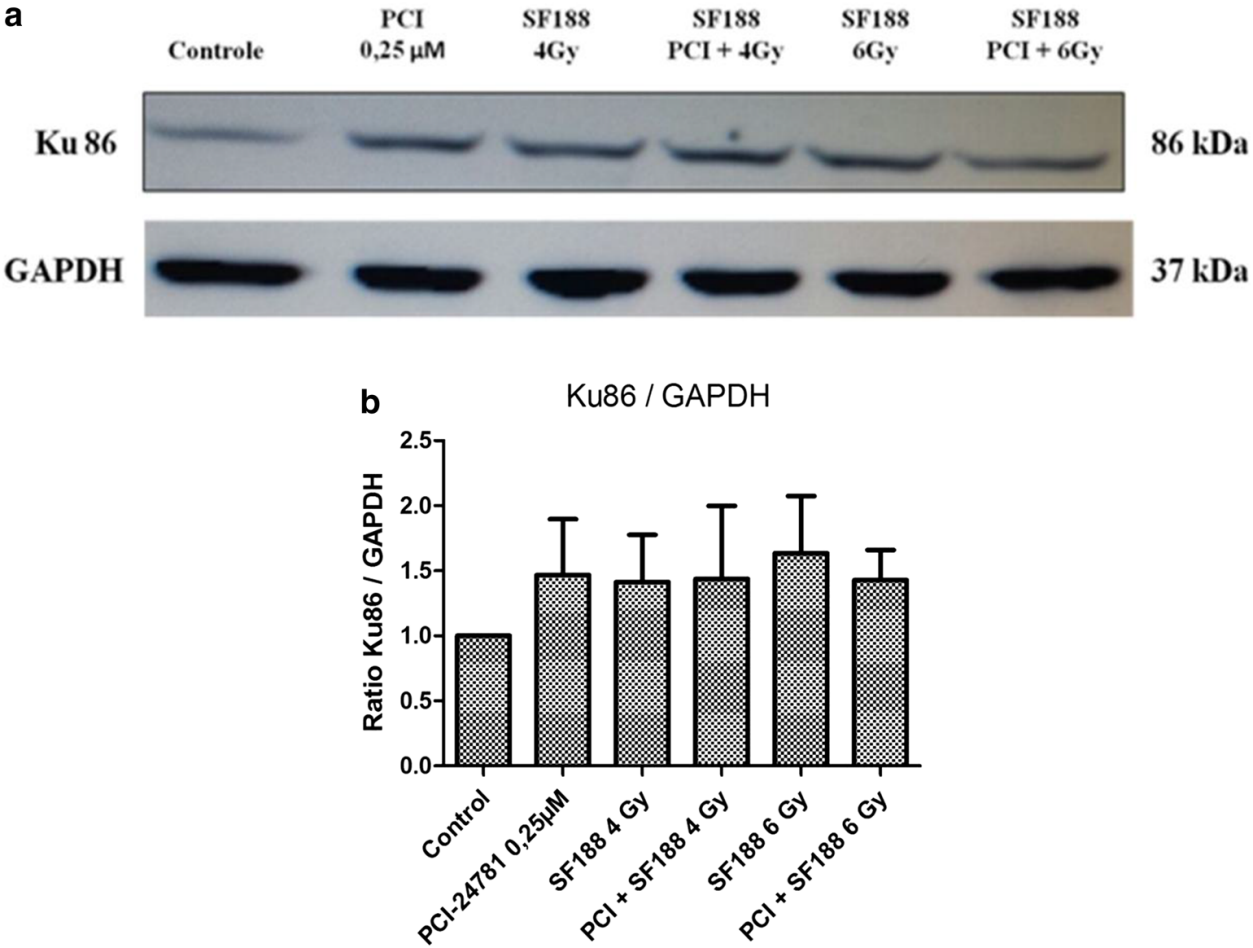
PCI-24781 alters the expression of the Ku86 protein. **a** PCI-24781 reduces the expression of the Ku86 protein, important for the repair of double-strand breaks caused by irradiation through the nonhomologous end joining (NHEJ) pathway. **b** Ratio between the Ku86 protein and endogenous GAPDH

**Fig. 9:**
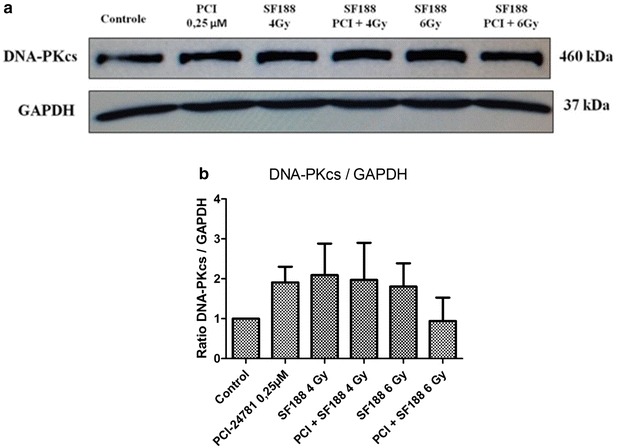
PCI-24781 alters the expression of the DNA-PKcs protein. **a** PCI-24781 reduces the expression of the DNA-PKcs protein, important for the repair of double-strand breaks caused by irradiation through the nonhomologous end joining (NHEJ) pathway. **b** Ratio between the DNA-PKcs protein and endogenous GAPDH

**Fig. 10:**
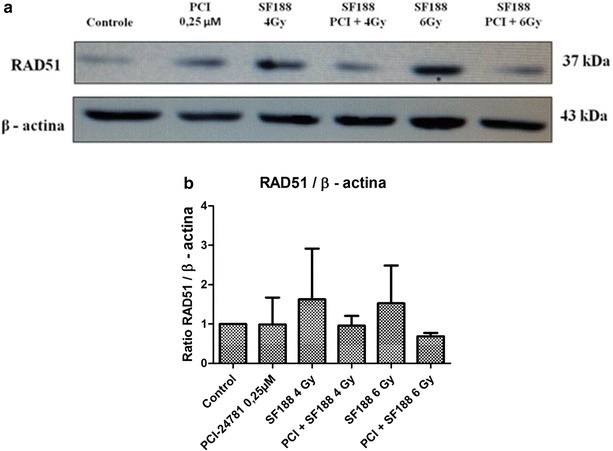
PCI-24781 alters the expression of the RAD51 protein. **a** PCI-24781 reduces the expression of the RAD51 protein, important for the repair of double-strand breaks caused by irradiation through the nonhomologous end joining (NHEJ) pathway. **b** Ratio between the RAD51 and endogenous β-actin

The results showed that PCI-24781 was competent in reducing the efficiency of the SF188 line in diminishing the DSB induced by irradiation, with a reduction of the expression of the proteins tested. For the Ku 70, Ku 86 and DNA-PKcs proteins there was only a small difference between treatment with PCI-24781 alone or in combination (Figs. 7, 8, and 9 respectively). The most evident decrease was observed for the RAD51 protein, present in the HR pathway. For all proteins, treatment with PCI-24781 followed by 6 Gy of irradiation demonstrated the greatest inhibition of the repair pathway (Fig. 10).

## Discussion

Over the last few years, the association between HDACs and carcinogenesis has increased the interest in the use of HDACi as antitumor agents. HDACi are new anticancer agents that induce tumor cell death and differentiation, chromatin decondensation and arrest of the cell cycle [33].

Uncontrolled cell proliferation is a strong characteristic of aggressive tumors, including GBM [34]. In the present study, the PCI-24781 inhibitor was found to cause efficient inhibition of cell proliferation and increased cell death by apoptosis in both the SF188 and KNS42 cell lines. Similar effects of inhibition of proliferation and induction of apoptosis have been reported in the literature for PCI-24781 and other HDACi, in separate treatments or in combination with other agents against neoplastic cell lines [35–40]. Some in vivo experiments have also been reported in the literature with the objective of investigating the possible mechanisms involved in the therapeutic effect of the HDACi PCI-24781 in the inhibition of cell proliferation in mice and on patient-derived xenografts [41, 42].

Both lines studied herein here have a mutation of the *TP53* gene, but several studies have demonstrated no significant difference in the response to treatment with PCI-24781 between *TP53*-proficient and *TP53*-deficient cell lines [35–39]. Dalvai and Bystricky demonstrated that the histone hyperacetylation caused by HDAC inhibitors is responsible for the antiproliferative effect of the inhibitors, which is probably due to specific changes in the expression of genes involved in the progression from the G1 to the S phase [43]. Several studies have also reported that the expression of important proteins involved in the apoptosis pathway such as Bad, Bax, caspase 3, caspase 8, Fas, p21, TP53 and DR4 was high after treatment with PCI-24781, suggesting that this HDACi activates both the intrinsic and extrinsic pathways of apoptosis [36, 37, 39, 44].

Regarding the clonogenic capacity, we observed that after treatment there was a significant reduction of colony formation in both lines. Several studies have detected results similar to those obtained here with PCI-24781 and various other HDACi in separate or combined treatments of neoplastic cell lines [35, 39, 42].

Clinically, two HDACi inhibitors approved by the FDA, suberoylanilide hydroxamic acid (SAHA) and valproic acid (VPA), were used in phase I studies conducted on children with primary or relapsing CNS tumors. The treatments were carried out using the inhibitor alone or in combination with chemotherapy and were well tolerated in all studies [44–49].

Radiotherapy is an important part of the treatment of pediatric GBM. Strategies increasing the efficiency of radiotherapy and permitting a reduction of toxic effects on normal tissue are important. In this respect, in the current study we also investigated the radiosensitizing potential of PCI-24781 using clonogenic assays, which showed that treatment with the inhibitor significantly increased the sensitivity to radiation of both lines.

Other studies have also assessed the radiosensitizing effect of PCI-24781 and of other HDACi based on clonogenic capacity in tumors other than GBMs. Chen et al. (2009) assessed the radiosensitizing capacity of the HDACi VPA on two colon tumor cell lines, one of them with a p53 mutation and the other without a mutation. The line with no *TP53* mutation showed a greater reduction of colony forming capacity, suggesting that *TP53* plays an important role in the modulation of the effect of HADCi [50]. Munshi et al. observed an accumulation of acetylation in the H4 histone after treatment with different HDACi, although the radiosensitizing effect on the lines was not influenced by this hyperacetylation, with this radiosensitizing mechanism provoked by HDACi being unknown [51].

Radiation remains an essential modality in the treatment of various brain tumors. Radiotherapy can be delivered alone or in combination with a radiosensitizer agent, in order to enhance the efficacy of irradiation. As revised by Harasaki and Waziri (2012) [52], the conceptual basis for radio sensitization includes at least five different mechanisms: spatial cooperation, biologic cooperation, cytotoxic enhancement, temporal modulation and protection of normal tissues. The number of different molecules that may be used concomitant to radiotherapy still is evolving. Besides some classical agents in clinical use (temozolomide, topotecan, irinotecan, vinka alkaloids and others), more recently biological modifiers such as bevacizumab, lenalidomide, sorafenib, erlotinib and dasatinibe have been proposed to display radiosensitizing properties for clinical investigation for malignant glioma [52]. Many of these compounds (particularly temozolomide and topoisomerase I an II agents) exert their radiosensitizer activity via double strand breaks (DSBs). The double-strand breaks are the major injuries occurring in response to irradiation and their repair is fundamental in order to determine radiosensitivity. In this respect, the HDAC inhibitors specifically target HDACs, a pivotal element in the process of condensation and transcription of the DNA, abolishing the DNA/protein response caused by ionizing radiation. This molecular mechanism of action supports the putative role the HDAC inhibitors as potential cytotoxic and radiosensitizing mediators [53–56]. Indeed, our study depicted a moderate cytotoxic effect of PCI-24781 on cell proliferation and apoptosis, particularly at higher drug concentrations. Although not contemplated in this study design, the role of PCI-24781 immediately prior and following to cell irradiation deserves further evaluation.

In the present study, in order to validate the radiosensitizing effects observed, we investigated the effect of PCI-24781 on the response of the SF188 line in terms of the repair of irradiation damage, affecting proteins of two important repair pathways, i.e., homologous recombination (HR) and non-homologous end joining (NHEJ). The results showed that PCI-24781 affected the expression of important proteins of both the HR pathway (the RAD51 protein) and of the NHEJ pathway (the KU70, Ku86 and DNA-PKcs proteins), leading us to believe that the inhibitor proved to be competent in reducing the efficiency of the SF188 cell line for the repair of irradiation-induced DSB. RAD51 is an important repair protein responsible for mediating the search of a homologous DNA sequence to be used as a model and for the formation of molecular joining between the damaged and the undamaged strands [57]. It has been reported that high expression levels of these proteins are related to resistance to radiotherapy and chemotherapy and therefore low levels of these proteins responsible for DSB repair increase the radiosensitizing effect of the cells [57–60].

The present study was the first to assess the effect of the HDACi PCI-24781 on the expression levels of the proteins involved in double-strand DNA repair in pediatric GBM lines.

Previous studies of colon and gastric tumor lines and of xenographic models have also observed that the level of expression of the RAD51 protein is drastically reduced after treatment with PCI-24781, leading to inhibition of DSB repair by homologous recombination [38, 53, 54]. Several other authors have also investigated the radiosensitizing potential of various HADCi in terms of their capacity to reduce the repair of damage in different tumor lines, significantly reducing the expression of RAD51, an important protein for the HR repair pathways [53, 61], and of Ku86, Ku70 and DNA-PKcs, important proteins for the NHEJ repair pathway [54, 62, 63].

Despite the great interest in combining HDAC inhibitors with radiation as a form of clinical strategy for tumor treatment, the exact molecular mechanism of the radiosensitizing effect of these inhibitors is still not well known. HDAC inhibition facilitates the relaxation and opening of chromatin, rendering it more sensitive to radiation damage and also modifying the transcription of various genes, with these two processes playing an important role in the regulation of radiosensitivity. In addition, transcriptionally active genes have proved to be more sensitive to DNA damage produced by ionizing radiation, thus forming a favorable antitumor interaction between HDAC inhibitors and radiation. An explanation for this increased response to radiation following HDACi treatment may be the effect of these inhibitors on the DNA repair processes [14, 63–65].

The present study demonstrates that the HDACi PCI-24781 can reduce the ability of the pediatric GBM line to repair radiation-induced DNA damage, increasing the radio sensitization of the line.

## Conclusion

In conclusion we demonstrated that the HDAC inhibitor PCI-24781 reduced proliferation and clonogenic capacity and increased apoptosis of pediatric GBM cell lines. In addition the HDACi PCI-24781 demonstrates high radiosensitizing activity by reducing the capacity of the cells to repair DNA damage induced by radiation by altering the expression of nonhomologous proteins (NHEJ). Thus, PCI-24781 can be considered to be an agent with a good potential for studies of in vivo models of pediatric GBM treatment.

## Abbreviations

GBM: glioblastoma
CNS: central nervous system
HDACi: histone deacetylase inhibitor
HR: homologous recombination
NHEJ: non-homologous end joining
HATs: histone acetyltransferases
PI: propidium iodide
SAHA: suberoylanilide hydroxamic acid
VPA: valproic acid
DMSO: dimethylsulfoxide
